# Optimized microwave pretreatment and ultrasound-assisted deep eutectic solvent extraction for efficient valorization of *Citrus paradisi* peel bioactives

**DOI:** 10.1186/s40643-026-01137-x

**Published:** 2026-09-17

**Authors:** Saira Zumar, Faiza Imtiaz, Abdulrahman A. Almehizia, Ahmad A. Assiri, Arfaa Sajid, Qaisar Manzoor, Syed Tauseef Saeed

**Affiliations:** 1 Department of Chemistry, The University of Lahore, Lahore, 54000 Pakistan; 2 Department of Pharmaceutical Chemistry, College of Pharmacy, King Saud University, 11451 Riyadh, Saudi Arabia; 3 Department of Pharmacognosy, College of Pharmacy, Najran University, Najran, Saudi Arabia; 4 Department of Mathematics, The University of Lahore, Lahore, 54000 Pakistan; 5 Department of Mathematics, Northwestern Polytechnical University, Xi’an, China

**Keywords:** Deep eutectic solvents (DES), *Citrus paradisi* peel, Microwave-pretreated ultrasound-assisted extraction (MP-UAE), Polyphenol extraction, Response surface methodology (RSM), Glycerol–glucose solvent system

## Abstract

**Abstract:**

This study presents a sustainable approach for recovering bioactive compounds from grapefruit (*Citrus paradisi*) peel using a glycerol–glucose-based deep eutectic solvent as a green extraction medium. Spectroscopic characterization confirmed the formation of stable hydrogen-bonded eutectic networks within the solvent systems. Response surface methodology (RSM) based on a Box–Behnken design was employed to systematically investigate the effects of solvent concentration, solvent-to-solid ratio, and extraction time on phytochemical recovery and antioxidant activity. The extraction process was successfully optimized using RSM, with the optimum conditions identified as 74% water-content Aq-DES, 20 min sonication time, and a solvent-to-solid ratio of 35 mL/g. Under these conditions, the optimized extract exhibited total phenolic and flavonoid contents of 86.33 mg gallic acid equivalents (GAE)/g DW and 5.93 mg rutin equivalents (RE)/g DW, respectively, along with strong antioxidant activities, achieving 87.40% scavenging of 2,2-diphenyl-1-picrylhydrazyl (DPPH) radicals and 55.09% scavenging of 2,2′-azino-bis(3-ethylbenzothiazoline−6-sulfonic acid) (ABTS) radicals. The developed RSM models demonstrated strong statistical adequacy and reliable predictive performance. Furthermore, the optimized extracts exhibited notable antibacterial activity against *Staphylococcus aureus* and *Salmonella typhi*, producing inhibition zones of 21 and 20 mm, respectively, in the undiluted extract. Overall, the findings demonstrate that glycerol–glucose deep eutectic solvent based microwave pretreated ultrasound-assisted extraction provides an efficient, rapid, and environmental friendly strategy for valorizing grapefruit peel waste into high-value antioxidant and antimicrobial products, with promising potential for applications in the food and pharmaceutical industries.

**Graphical abstract:**

**Figure:**
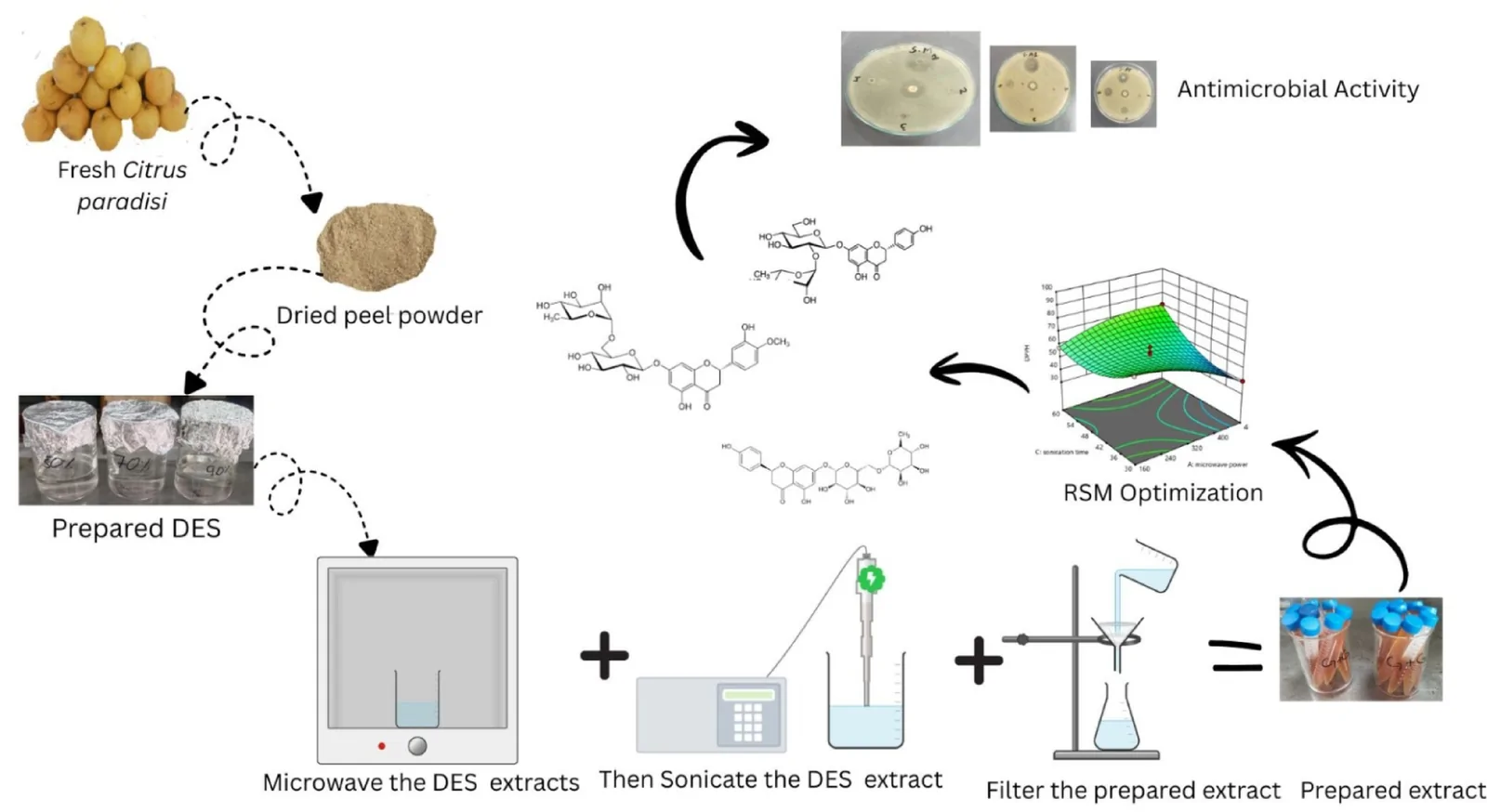

## Introduction

Deep eutectic solvents (DESs) are a new class of environmentally safe solvents with a wide range of potential uses in the food, chemical, and pharmaceutical industries (Ali and Ahmed 2023). DES based solvents, which are created by hydrogen-bond interactions between a hydrogen-bond donor (HBD) and a hydrogen-bond acceptor (HBA), have melting points that are significantly lower than those of their constituent parts, allowing liquid formation under mild heating. Their advantages, including low volatility, tunable polarity, high solubilization capacity, and biodegradability, have positioned DESs as promising alternatives to conventional solvents and, in many cases, to ionic liquids (Imtiaz et al. 2024). Owing to their structural flexibility and ability to incorporate natural or bio-derived constituents, DESs offer a highly adaptable solvent platform for sustainable extraction and processing technologies.

Deep Eutectic Solvents (DES) based on naturally-occurring metabolites have been of great interest in recent times due to their potential use in sustainable extraction of bioactive compounds from plant-based sources (Ivanović et al. 2022; Shu et al. 2026a, b, c, d, e). NaDES that involve the use of metabolites including sugars, polyols, organic acids, and amino acids possess tunable polarity, high hydrogen bonding capability, low vapor pressure, and good biocompatibility (Fernandes and Buoro 2025; Vidić et al. 2025). In particular, glycerol-glucose NaDES stands out as a very interesting mixture due to the fact that the hydroxyl groups in glycerol and glucose molecules can interact with the hydroxyl groups in the cell wall structure of the plants and phenolic compounds to facilitate their solubility and recovery (Ramon et al. 2023; Freitas et al. 2025; Shu et al. 2026a, b, c, d, e).

Peels from *Citrus paradisi* (grapefruit) are a common agro-industrial by-product that is high in bioactive components like flavonoids, phenolic acids, and other polyphenols (Xie et al. 2025). Grapefruit peel is a useful raw material for nutraceutical, food, cosmetic, and pharmaceutical applications because of these phytochemicals strong antioxidant, anti-inflammatory, and cardioprotective qualities (Andrade et al. 2022). Despite their high content of valuable compounds, large quantities of citrus peels are typically discarded or underutilized, highlighting the importance of developing efficient, low-cost, and environmentally safe extraction technologies. Green solvent systems such as DESs offer a strategic approach to valorizing this biomass while aligning with circular-economy principles.

Extraction efficiency of phytochemicals largely depends on solvent properties, including polarity, viscosity, acidity, and hydrogen-bonding capacity (Kumar et al. 2023; Sajid et al. 2025). Despite their effectiveness, conventional organic solvents like methanol, ethanol, and acetone raise issues with toxicity, environmental persistence, and high energy requirement (Joshi and Adhikari 2019). In contrast, DESs have demonstrated enhanced solubilization of polar and semi-polar plant metabolites due to their extensive hydrogen-bond networks and tunable physicochemical characteristics (tul Kubra et al. 2023; Imtiaz et al. 2024; Shu et al. 2025a, b). Recent research has shown that DES-based extraction can achieve higher yields of total phenolic content (TPC) and antioxidant capacity compared to conventional solvents, while offering improved safety and sustainability. Their unique interactions with phenolic structures make DESs particularly suitable for extracting hydrophilic compounds from citrus matrices (Meng et al. 2025; Shu et al. 2026a, b, c, d, e).

Ultrasound-assisted extraction (UAE) is a very efficient and popular technique for extracting valuable bioactive compounds from plant materials (Rueangsri et al. 2025; Shu et al. 2025a, b; Shu et al. 2026a, b, c, d, e). The cavitation effect generated by ultrasonic waves enhances cell disruption and facilitates the release of intracellular bioactive compounds into the extraction medium (Tahir et al. 2024). MP-UAE has enhanced this method by coupling the rapid cell disintegration with microwave pretreatment and the application of cavitation effects of ultrasound (Shu et al. 2026a, b, c, d, e). Cells in the plant materials can be easily degraded by microwave and ultrasound may improve the penetration of solvent and mass transfer (Liu et al. 2022; Singla et al. 2023). This technique will therefore help to increase the yield of phenolic compounds, flavonoids and other important phytochemicals with reduced extraction times and reduced solvent usage. The use of green solvents like deep eutectic solvents (DESs) in these hybrid methods is a sustainable way to carry out the extraction process compared to conventional methods (Altarjami 2025).

Optimization of extraction conditions is crucial for maximizing recovery of bioactive compounds and ensuring process efficiency. Response surface methodology (RSM), particularly the Box–Behnken design (BBD), is widely applied for modeling and optimizing multivariable extraction systems due to its statistical efficiency and reduced number of experimental runs (Tourabi et al. 2025; Imtiaz et al. 2026). RSM enables the identification of significant factors, elucidation of interaction effects, and prediction of optimal conditions for target responses such as TPC and antioxidant activity. Employing BBD in DES-based extraction offers a systematic approach to refining parameters such as solvent composition, extraction temperature, time, and solid–liquid ratio, thereby improving overall extraction performance (Iftikhar and Ahmed 2025).

The present study focused on the recovery of phenolic compounds from *Citrus paradisi* peels using a glycerol–glucose natural deep eutectic solvent (NaDES). The glycerol–glucose system was selected for further investigation based on its favorable extraction performance and was subsequently optimized using response surface methodology coupled with a Box–Behnken design (RSM–BBD) to maximize phenolic recovery. Unlike our previous DES-based extraction studies, the present work investigates a glycerol–glucose NaDES system specifically for the valorization of grapefruit peel and integrates its optimized extraction with evaluation of both antioxidant and antibacterial properties, thereby extending the application of DES-based extraction toward the multifunctional valorization of citrus processing waste. Overall, the study aimed to develop an environmentally friendly, efficient, and statistically optimized glycerol–glucose NaDES-based extraction approach for the valorization of grapefruit peel waste.

## Materials and methods

### Sample preparation

Fresh *Citrus paradisi* (grapefruit) fruits were obtained from the local market. The peels were manually separated, washed, and weighed. The fresh peel weight was recorded as 1.86 kg. The peels were shade‑dried for 4–8 days until constant weight was achieved, resulting in 1.01 kg of dried peel. The dehydrated peels were milled into a uniform powder to be used in subsequent extraction processes.

### Synthesis of deep eutectic solvents

Glucose–glycerol NaDES (1:2 molar ratio) was prepared by mixing density-corrected glycerol (146.3 mL, corresponding to 2 mol) with glucose (180.16 g, 1 mol). The mixture was heated at 80 °C under continuous stirring until a clear and homogeneous liquid was obtained. The prepared NaDES was subsequently allowed to equilibrate at room temperature for 24 h before use.

Three aqueous DES (Aq-DES) formulations were prepared by varying the water content of the DES solutions. The formulations were designated as 50%, 70%, and 90% Aq-DES, where the percentage denotes the volume percentage of distilled water in the final solution. Accordingly, the 50% Aq-DES solution was prepared by mixing 50 mL of DES with 50 mL of distilled water, the 70% Aq-DES solution by mixing 30 mL of DES with 70 mL of distilled water, and the 90% Aq-DES solution by mixing 10 mL of DES with 90 mL of distilled water.

#### Characterization of DES

A variety of analytical methods were used to analyze the synthesized DES in order to verify their production and assess their chemical and structural characteristics. The optical characteristics of the DES were evaluated using UV–VIS spectroscopy (CECIL 7400CE AQUARIUS, Cecil Instruments, Cambridge, UK), with absorption spectra collected in the 150–800 nm range. The functional groups were identified using Fourier Transform Infrared (FT-IR) spectroscopy (BRUKER ALPHA-2, Bruker Corporation, Germany), which scanned the spectrum range of 400–4500 cm⁻^1^ (Imtiaz et al. 2024). The FTIR analysis was performed using the FTIR facility of the Department of Chemistry, The University of Lahore.

### Preliminary studies

**Fig. 1:**
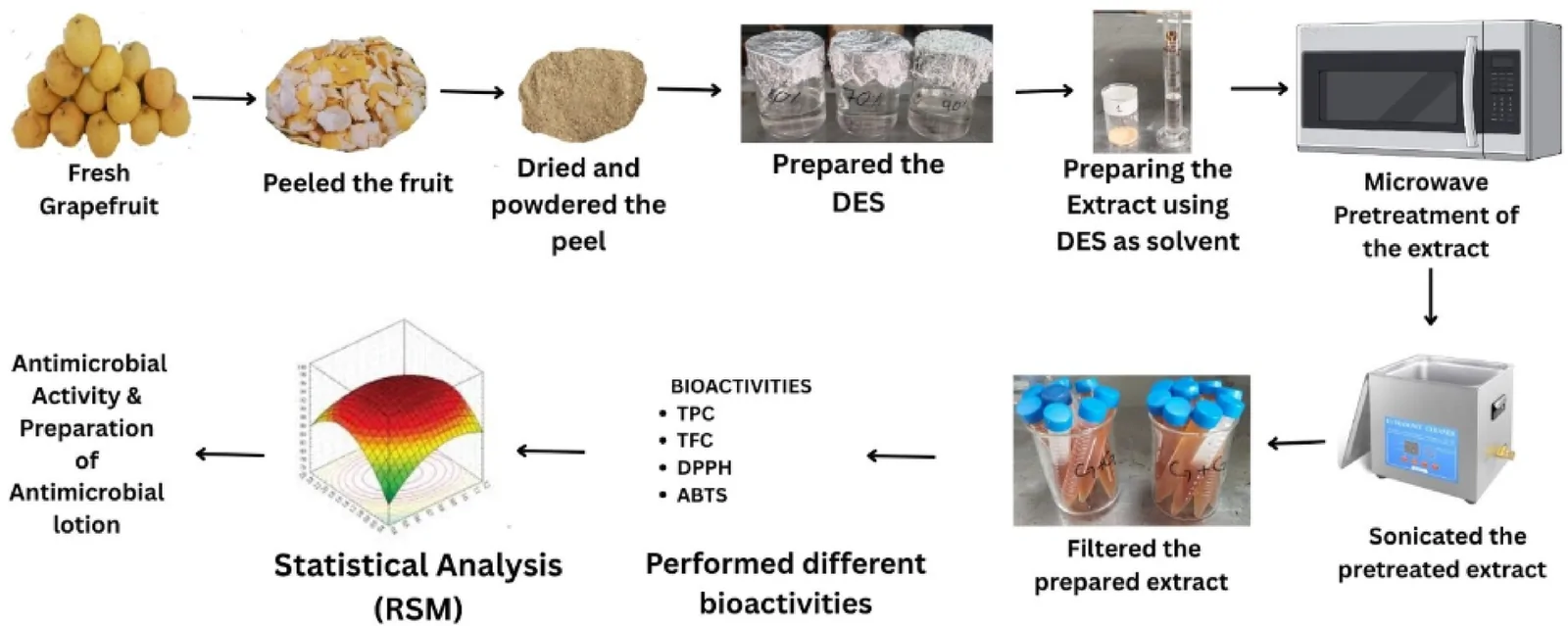
Flow chart of the extraction process

For the microwave pretreated ultrasound-assisted extraction (MP-UAE_DES_), extracts were prepared using 70% formulations of DES (Fig. 1). A measured volume of 25 mL of the DES was combined with 1 g of powdered citrus peel to obtain a solid-to-solvent ratio of 1 g per 25 mL. the experiments were repeated in triplicates. An SSR of 25 mL/g was initially employed for preliminary screening to evaluate the extraction effectiveness of the glucose–glycerol DES. The mixtures were initially subjected to microwave-assisted extraction for 1 min at 480 W using household microwave oven (LG Intellowave Sensor model MS-3242DPS, LG Electronics, South Korea), followed by 30 min of sonication to enhance solvent penetration and compound release. After treatment, the samples were filtered, and the resulting extracts were collected for subsequent TPC and TFC analyses.

### Total phenolic and flavonoid content

Our previously described approach was used to measure the total phenolic and flavonoid contents (Imtiaz et al. 2023). TPC was determined by mixing 100 μL extract with water and Folin–Ciocalteu reagent, followed by Na₂CO₃ addition, incubation (30 min at 40 °C), and absorbance measurement at 765 nm. Results were expressed as mg GAE/g DW (dried weight) using a gallic acid calibration curve (50–500 μg/mL; y = 0.0009x + 0.0112, R^2^ = 0.99).

TFC was measured by reacting 300 μL of extract with aqueous methanol, adding NaNO₂, AlCl₃, and NaOH, and then measuring absorbance at 506 nm. A rutin calibration curve (50–250 μg/mL; y = 0.003x + 0.04, R^2^ = 0.998) was used to express the results as mg RE/g DW. A DES-only matrix blank was used to correct the background response, and its contribution was subtracted from the corresponding TPC and TFC measurements.

### Antioxidant assays

DPPH and ABTS assays (DPPH-RS and ABTS-RS respectively) were used to measure antioxidant activity in accordance with previously published procedures without any additional modifications (Imtiaz and Ahmed 2026).

### Design of experiment (DOE)

In order to examine the impact and interplay of important processing variables on the extraction efficiency of the synthesized DES system, the extraction process was optimized using a Box–Behnken design (BBD). Three independent factors were evaluated: Aq-DES (50–90% aqueous solutions), sonication time (20, 30, and 40 min), and solvent to solid ration (mL/g). The BBD produced 17 experimental runs in total, which minimized the number of necessary experiments while enabling the evaluation of linear, quadratic, and interaction effects on the extraction response.

For each experimental run, 1 g of powdered citrus peel was combined with the Aq-DES water content (%) according to the BBD matrix. The mixtures were exposed to microwave treatment for 1 min, followed by sonication for the specific duration assigned within the design. After extraction, all samples were vacuum-filtered to remove plant residues, and the resulting filtrates were collected in sterile, capped centrifuge tubes for further phytochemical and analytical evaluations (Imtiaz, Ahmed et al. 2026).

### Analysis of the data

Design-Expert software (Version 12, MN, US) was utilized for statistical analysis, model fitting, and experimental design. All calculations were made three times for optimization studies, and the statistical mean was computed using SD (standard deviation). The relevance of the model terms was assessed using analysis of variance (ANOVA). Terms were deemed significant if their p-values were less than 0.05 (Imtiaz et al. 2024).

### Antimicrobial activities

The extraction of the plant material for antimicrobial testing was performed using the optimized DES system. Initially, 25 mL of the 70% Aq-DES solution was mixed with 1 g of plant peel powder to obtain a 1:25 (w/v) extraction ratio. The mixture was subjected to the standardized extraction conditions and subsequently filtered to remove solid residues. The resulting crude extract was used to prepare two serial dilutions, with 70% Aq-DES employed as the diluting solvent. Each successive dilution was prepared by mixing equal volumes of extract and solvent, yielding three test concentrations: the undiluted extract, the first serial dilution (1:1), and the second serial dilution (1:2). These dilutions were then used for antimicrobial assays. Antimicrobial activity of the optimized DES extract was evaluated using the agar well diffusion assay. The bacterial strains were obtained from the Institute of Molecular Biology and Biotechnology (IMBB), University of Lahore, Pakistan, and were used for the antibacterial assays. Two bacterial strains were selected for analysis: *Staphylococcus aureus* (SA) and *Salmonella typhi* (SM), with vancomycin (VA30) and ciprofloxacin (CIP5) serving as their respective reference antibiotics. Blood agar medium was prepared by dissolving 7.4 g of dehydrated medium in 200 mL of distilled water, followed by sterilization through autoclaving. The molten medium was poured into sterile Petri plates and allowed to solidify under aseptic conditions for 3–5 h. Once solidified, wells were aseptically created in the agar surface and appropriately labeled. The optimized DES extracts were carefully dispensed into the wells, while standard antibiotic discs were placed at the center of the plates as positive controls. The inoculated plates were incubated at 37 °C for 24 h to allow microbial growth and diffusion of the test extracts. Following incubation, the diameter of the inhibition zones was measured and compared with the zones produced by the standard antibiotics to assess the relative antimicrobial efficacy of the extracts .

## Results and discussion

A preliminary screening study was conducted to evaluate the effectiveness of the glucose-glycerol (Glu–Gly) natural deep eutectic solvent (NaDES) for the extraction of bioactive compounds from *Citrus paradisi* peel. The screening was performed by assessing the total phenolic content (TPC) and total flavonoid content (TFC) of the resulting extract. The Glu–Gly system yielded 82.997 mg GAE/g DW dried powder (DP) of TPC and 18.563 mg RE/g DW of TFC. The result demonstrated favorable recovery of phenolic and flavonoid compounds from the waste peels, indicating its effective interaction with the phytochemical constituents (Hilali et al. 2022; Santos et al. 2025). The high hydroxyl functionality of glycerol and glucose provides an extensive hydrogen-bonding network that can facilitate the solubilization of polar and moderately polar phytochemicals. Based on its favorable TPC and TFC recovery during the preliminary screening, the Glu–Gly NaDES was selected as the extraction solvent for subsequent optimization using response surface methodology (RSM) and Box–Behnken design (BBD). This preliminary assessment therefore provided the basis for selecting Glu–Gly as a suitable green extraction medium for further process optimization.

### UV visible analysis of synthesized DES

**Fig. 2:**
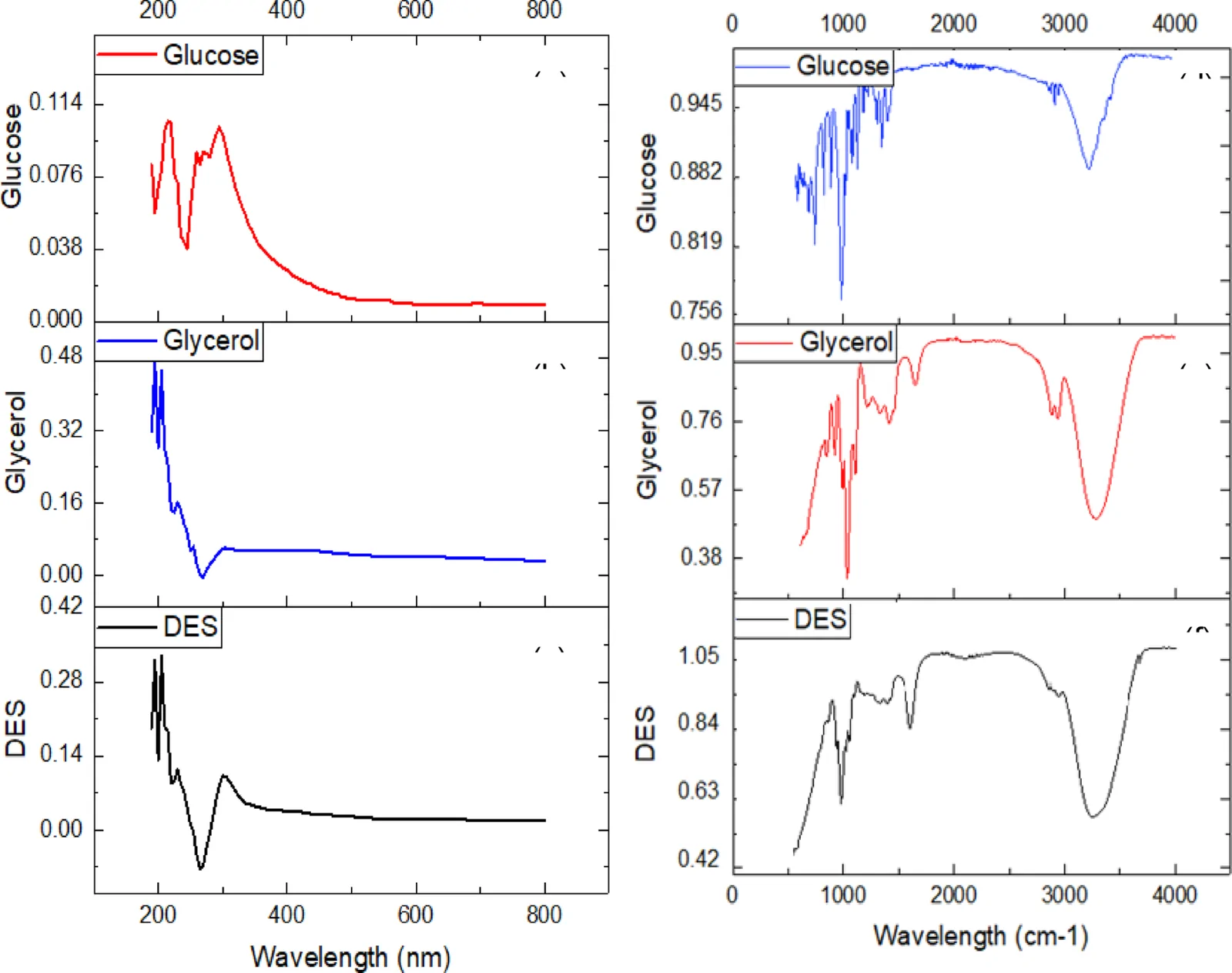
UV–visible and FTIR spectra of the synthesized glucose-glycerol NaDES: UV–Vis spectra of **a** glucose, **b** glycerol, and **c** glycerol–glucose NaDES; FTIR spectra of **d** glucose, **e** glycerol, and **f** glucose-glycerol NaDES

The UV–Visible absorption spectra of of synthesized Glu-Gly DES is presented in Fig. 2a–c. The UV–Vis profiles of the individual components, glucose and glycerol, exhibit distinct absorption characteristics that reflect their molecular electronic environments. Glucose shows two identifiable absorption features in the 200–230 nm region, which are attributed to n→σ* transitions of its hydroxyl groups and to electronic transitions associated with the small fraction of its open-chain aldehyde form (Fig. 2a).

Glycerol exhibited a pronounced absorption in the deep-UV region, particularly below approximately 220 nm, followed by a sharp decrease in absorbance with increasing wavelength. A weak residual absorbance was observed over the 300–800 nm range; however, its magnitude was substantially lower than that in the deep-UV region, indicating that glycerol has limited absorption in the near-UV and visible regions (Fig. 2b). The observed spectrum is consistent with the UV–Vis behavior of glycerol reported in the literature, where the characteristic absorption around 222 nm has been utilized for glycerol determination (Glavcheva-Laleva et al. 2015).

Upon formation of the glucose-glycerol DES, the resulting spectrum no longer appears as a simple mathematical combination of the two individual profiles. Instead, the DES spectrum shows characteristic broadening of the glucose-derived features and a slight shift of the absorption shoulder near 220–240 nm, superimposed on the strong low-wavelength absorption inherited from glycerol (Fig. 2c). These modifications indicate the formation of an extensive hydrogen-bonding network within the DES, which perturbs the local electronic transitions of glucose and enhances band broadening through altered solvation dynamics and increased structural disorder. The absence of new absorption features at higher wavelengths confirms that DES formation proceeds without the generation of new chromophores, supporting the interpretation that the mixture undergoes physical complexation rather than chemical reaction.

### FTIR analysis of synthesized DES

Figure 2d–f shows the FTIR spectra of the synthesized DES and its corresponding individual components, highlighting the interactions between constituents.

For glycerol, FTIR analysis revealed a broad O–H stretching vibration region in the range of ~3200–3500 cm⁻^1^, which is common to hydroxyl groups participating in hydrogen bonding, along with C-H stretching vibration at 2900 cm⁻^1^ and C-O stretching bands in the ~1000–1150 cm⁻^1^ region. For glucose, there was also a broad O–H stretching vibration in the range of ~3200–3400 cm⁻^1^, C-H stretching at 2900 cm⁻^1^ and characteristic C-O/C–O–C stretching vibration in the fingerprint region (Ivanov-Omskii 2014, Barretto et al. 2026). After obtaining glucose-glycerol NaDES, there were significant changes in O–H stretching vibration region with respect to broadening of the bands and shift of the bands, which indicate the modification of hydrogen bonding interaction of the hydroxyl groups (Ahmed et al. 2026). There were also changes in the C-O/C–O–C region, as a consequence of interactions of functional groups of glucose with glycerol. Based on the above information, it is evident that hydrogen-bond interactions between the molecules and formation of hydrogen bonded network took place.

### RSM based extraction optimization

On the basis of preliminary results, glucose-glycerol DES was used for optimization studies. MP-UAE_DES_ based extraction of *C. paradisi* using prepared DES was optimized to develop a process for the extraction of bioactive compounds.

The design of the experiment and its results are shown in Table 1.

**Table 1:** Box–Behnken design (BBD) outcomes showing different independent microwave parameters and their corresponding responses for MP-UAE_DES_ extraction

Run	Factor 1 SSR (mL/g)	Factor 2 Aq-DES (%)	Factor 3 time (min)	Response 1 TPC (mg GAE/g DW)	Response 2 TFC (mg RE/g DW)	Response 3 DPPH-RS (%)	Response 4 ABTS-RS (%)
1	25	50	20	66.58 ± 1.12	4.39 ± 0.12	91.26 ± 0.16	47.45 ± 2.01
2	25	70	30	63.58 ± 1.09	5.82 ± 0.71	64.92 ± 0.28	43.76± 2.87
3	25	70	30	75.46 ± 0.84	6.15 ± 0.11	67.73 ± 0.41	44.75± 2.24
4	35	70	20	86.41 ± 1.47	5.67 ± 0.97	91.16 ± 0.87	53.86± 2.36
5	25	70	30	72.10 ± 0.76	5.71 ± 0.49	67.47 ± 0.71	47.87± 1.91
6	25	90	20	62.88± 0.81	5.15 ± 0.88	69.51 ± 0.71	42.77± 1.89
7	25	50	40	62.77 ± 1.82	7.19 ± 0.71	61.98 ± 0.94	49.57± 2.36
8	15	90	30	49.79 ± 1.11	5.53 ± 1.18	48.36 ± 2.01	34.88± 2.59
9	35	90	30	76.70 ± 0.99	6.67 ± 0.28	70.5 ± 0.80	42.49± 2.33
10	35	70	40	86.72 ± 0.91	7.63 ± 0.55	57.16 ± 0.39	50.02± 2.74
11	35	50	30	85.08± 1.28	6.29 ± 0.65	83.94 ± 1.65	49.15± 2.09
12	25	90	40	70.94 ± 1.72	7.33 ± 0.38	42.75 ± 0.88	38.52± 3.01
13	15	50	30	46.23 ± 1.71	5.15 ± 0.87	64.42 ± 2.07	41.07± 3.11
14	25	70	30	70.94 ± 1.12	6.23 ± 0.93	68.66 ± 0.63	44.19± 2.64
15	25	70	30	71.58 ± 1.82	6.71 ± 1.08	66.14 ± 0.11	44.75± 1.82
16	15	70	40	47.89 ± 1.89	5.94 ± 0.93	49.91 ± 0.23	46.88± 1.98
17	15	70	20	49.09 ± 1.66	4.47 ± 1.00	74.97 ± 0.65	45.30± 1.06

The effects of various independent factors on to TPC, TFC and DPPH-RS and ABTS-RS was evaluated and is discussed below.

#### Effects of MP-UAEDES extraction on TPC

The highest TPC of 86.72 ± 2.01mg GAE/g DW was obtained using 70% Aq-DES, SSR of 35 mL/g, and a sonication time of 40 min while the minimum was obtained as 46.23 ± 1.71 mg GAE/g DW was obtained using 50% Aq-DES, SSR of 15 mL/g, and a sonication time of 30 min.

##### Effect of DES concentration

**Fig. 3:**
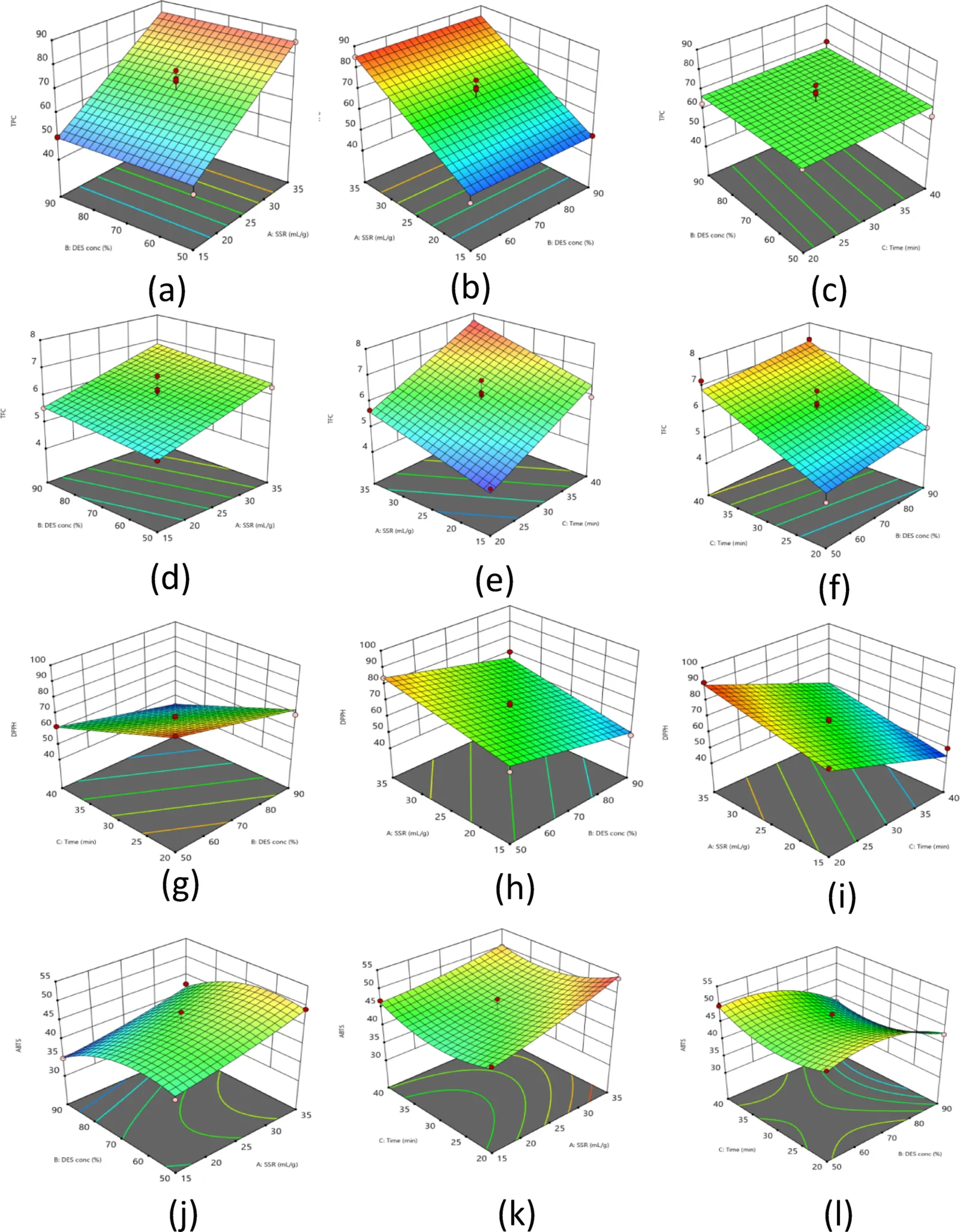
Microwave pretreated ultrasound-assisted extraction: 3D response surface plots of effects of different variables on (i) TPC (**a**–**c**), (ii) TFC (**d**–**f**), (iii) DPPH-Act (**g**–**i**), (iv) ABTS-Act (**j**–**l**)

Figure 3 shows effect of different input variables on responses as 3D response surface plots. The concentration of DES and its dilution with water significantly influenced the extraction efficiency of phenolic compounds from *Citrus paradisi* peel waste. Since a 90% Aq-DES corresponds to 90 mL water and only 10 mL DES, higher DES concentration values actually indicate a more diluted solvent system.The results show that TPC increased with increasing DES concentration up to the intermediate level (70%, corresponding to 70 mL water + 30 mL DES) and then slightly declined at 90%. At low dilution (50%, meaning 50 mL water + 50 mL DES), the extraction efficiency was lower, likely due to the high viscosity of the DES mixture which restricts mass transfer and solvent penetration into the plant matrix (Ling and Hadinoto 2022). Conversely, excessive dilution (90%) reduces solvent polarity and hydrogen-bonding ability, which are essential for efficient solubilization of phenolic compounds. Therefore, the moderate dilution level (70%) provided the optimal.

##### Effect of solvent-to-solid ratio (SSR) and extraction time

An increase in the ratio of solvent/sample from 15 to 35 mL/g showed a significant increase in TPC, probably owing to the better diffusion and transport of the solutes. The maximum TPC (86.72 ± 0.91 mg GAE/g DW) was observed at a ratio of 35 mL/g (Al-Farsi and Lee 2008). After a TPC of 30 min of extraction, there was a slight drop in TPC, which means that the extraction process had reached equilibrium and degradation might have occurred afterwards (Kidak and Ince 2006).

#### Effect of independent variables on total flavonoid content (TFC)

The highest TFC of 7.63 ± 0.55 mg RE/g DW was obtained using 70% Aq-DES, SSR of 35 mL/g, and a sonication time of 40 min while the minimum was obtained as 4.39 ± 0.12 mg RE/g DW was obtained using 50% Aq-DES, SSR of 25 mL/g, and a sonication time of 30 min.

##### Effect of DES concentration

TFC followed a similar pattern to TPC with respect to DES concentration. The extraction of flavonoids was lowest at 50% Aq-DES and highest at 70%, decreasing again at 90%. This trend suggests that the moderate dilution level facilitates an appropriate balance between solvent polarity and viscosity, enabling better solubilization of flavonoids (Shi et al. 2025; Ren et al. 2026). Overly concentrated DES (low water content) is too viscous, hindering diffusion, while excessive dilution (high water content) weakens the hydrogen bonding interactions between DES components and flavonoid molecules, reducing extraction efficiency (Karadendrou et al. 2025).

##### Effect of solvent-to-solid ratio (SSR) and extraction time

The increase in SSR increased the TFC, with the maximum being achieved at 35 mL/g, probably because of better contact of solvent and solute and mass transfer of flavonoids (Bi et al. 2013; Wang et al. 2019). TFC also increased until 30 min and stabilized or even decreased, probably because of the extraction equilibrium reached or compound degradation. Therefore, 30 min was a good compromise between TFC recovery and stability (Annegowda et al. 2010; Kim and Lee 2017).

#### Effect of independent variables on DPPH radical scavenging activity

##### Effect of DES concentration

The DPPH radical scavenging activity, reflecting the antioxidant potential of the extracts, was significantly affected by the DES concentration. The antioxidant activity increased from 50 to 70% Aq-DES and decreased at 90%. Excessive dilution reduces extraction efficiency, whereas moderate dilution lowers viscosity and improves solvent mobility, enhancing the recovery of antioxidant compounds (Ozturk et al. 2018; Wan Mahmood et al. 2019).

##### Effect of solvent-to-solid ratio (SSR) and extraction time

Increasing the SSR from 15 to 35 mL/g markedly enhanced DPPH inhibition, indicating that higher solvent volumes promote the release of antioxidant compounds. This trend was consistent with the increased TPC and TFC, as phenolic and flavonoid compounds are major contributors to antioxidant activity (Dabetić et al. 2020). Extraction time also showed a similar trend, with DPPH activity increasing up to 30 min before slightly declining at 40 min, possibly due to degradation of heat- or time-sensitive compounds. Thus, moderate extraction times favor both antioxidant recovery and compound stability.

#### Effect of independent variables on ABTS radical scavenging activity

##### Effect of DES concentration

The ABTS radical scavenging activity followed a similar trend to DPPH. The maximum activity was observed at 70% Aq-DES, while both lower (50%) and higher (90%) dilutions resulted in reduced antioxidant potential. This confirms that the solvent’s polarity and viscosity balance at 70% Aq-DES is ideal for the extraction of ABTS-reactive antioxidants from grapefruit peel waste.

##### Effect of solvent-to-solid ratio (SSR) and extraction time

A clear positive relationship was observed between SSR and ABTS inhibition, with higher solvent-to-solid ratios promoting greater recovery of antioxidant compounds. At lower SSR values, limited solvent availability may restrict mass transfer and reduce extraction efficiency (Tan et al. 2011; Ali and Ahmed 2023). Extraction time showed a similar trend to TPC and DPPH activity, with ABTS inhibition increasing up to 30 min before declining slightly at 40 min. This decrease may result from oxidation or degradation of sensitive phenolic compounds during prolonged extraction. Thus, approximately 30 min appears to provide a suitable balance between antioxidant recovery and compound stability (Chanioti and Tzia 2018; García-Roldán et al. 2023).

Overall, the extraction efficiency and antioxidant activity of *Citrus paradisi* peel extracts were strongly dependent on the three studied factors. Moderate DES dilution (70%), high solvent-to-solid ratio (35 mL/g), and extraction time of around 30 min yielded the best results for all response variables. These findings highlight the critical role of solvent composition and extraction parameters in optimizing the recovery of phenolic and flavonoid compounds using green DES-based extraction systems.

### Model fitting and optimization

MP-UAE_DES_ extraction demonstrates strong and reliable model performance for all evaluated responses, including TPC, TFC, DPPH-RS, and ABTS-RS (Table 2). TPC, TFC, and DPPH-RS followed linear models, while ABTS-RS was best described by a quadratic model. The models showed high coefficients of determination, with R^2^ values of 0.9013 for TPC, 0.9089 for TFC, 0.9658 for DPPH-RS, and 0.9593 for ABTS-RS, indicating that more than 90% of the variability in each response was explained by the models. The corresponding adjusted R^2^ values (0.8785–0.9580) further confirm good model adequacy with minimal overfitting.

**Table 2:** Fit statistics for the obtained results

MP-UAE_DES_ extraction				
Terms	TPC	TFC	DPPH-RS	ABTS-RS
	Linear	Linear	Linear	Quadratic
Lack of fit p value	0.4765	0.8486	0.0755	0.7731
Model p value	<0.0001	<0.0001	<0.0001	<0.0001
R^2^	0.9013	0.9089	0.9658	0.9593
Adjusted R^2^	0.8785	0.8879	0.9580	0.9070
Predicted R^2^	0.8395	0.8578	0.9314	0.8059
F-value	39.55	43.23	122.52	18.34
df	3	3	3	9

All models were highly significant, as reflected by model p-values < 0.0001, demonstrating strong statistical reliability of the fitted equations. In addition, the lack-of-fit p-values were non-significant (0.0755–0.8486), indicating an acceptable agreement between predicted and experimental data.

Equations (1–4) regression models describing the effect of independent variables on TPC, TFC, DPPH, and ABTS responses for microwave-assisted extraction using DES. Following ANOVA-based model reduction, only the statistically significant terms (p < 0.05) were retained in the final models.

1 $$ {\text{TPC - MP - UAE}}_{{{\mathrm{DES}}}} = { 67}.{34 } + {17}.{\mathrm{74A}} $$

2 $$ {\text{TFC - MP - UAE}}_{{{\mathrm{DES}}}} = { 6}.00 + 0.{\mathrm{6463A}} + {1}.0{\mathrm{5C}} $$

3 $$ {\text{DPPH - MP - UAE}}_{{{\mathrm{DES}}}} = { 67}.{11} + {8}.{\mathrm{14A}}{-}{8}.{\mathrm{81B}}{-}{14}.{\mathrm{39C}} $$

4 $$ {\text{ABTS - }}\;{\text{MP - UAE}}_{{{\mathrm{DES}}}} = { 45}.0{7} + {3}.{\mathrm{42A}} - {3}.{\mathrm{57B}}{-}{3}.{8}0{\mathrm{B}}^{2} + {3}.{\mathrm{31C}}^{2} $$

where A = SSR (mL/g), B = Aq-DES (%), C = time (min).

Overall, these findings confirm that MP-UAE_DES_ extraction is an efficient and statistically robust extraction approach for phenolics, flavonoids, and antioxidant activities, providing accurate predictions with low experimental error.

A numerical optimization process was performed using Design Expert software to determine the optimum conditions for MP-UAE_DES_ (Table 3). All input variables were kept “in range,” TPC was set to “maximize,” while TFC and antioxidant responses (DPPH-RS and ABTS-RS) were maintained “in range” to ensure balanced extraction performance. The optimal MAE conditions obtained were 74% Aq-DES, 20 min sonication time, and 35 mL/g SSR, with an overall desirability of 0.974. Validation experiments showed close agreement between predicted and observed values with low error rates, confirming the adequacy and reliability of the developed model in predicting response variables.

**Table 3:** Predicted and observed response values under the optimum extraction conditions for MP-UAE_DES_

MP-UAE_DES_	Aq-DES (%)	Sonication time (min)	SSR (mL/g)	TPC (mg GAE/g DW)	TFC (mg RE/g DW)	DPPH-RS (%)	ABTS-RS (%)	Desirability
Predicted	74	20	35	84.65	5.63	88.06	53.85	0.974
Observed				86.33	5.93	87.36	55.09	
Error rate %				1.98	5.33	-0.80	2.30	

While the maximum TPC observed experimentally was at 40 min in Run 10 (86.72 ± 0.91 mg GAE/g DW), the results of numerical optimization indicated 20 min as the optimum extraction time. Such differences can be attributed to the fact that, in RSM optimization, a multi-response approach has been used as opposed to the single-objective optimization whereby only TPC was optimized. Although 40 min gave the highest value of TPC individually, it showed comparatively low antioxidant activity, especially regarding DPPH radical scavenging activity (57.16 ± 0.39%). On the other hand, 20 min gave a relatively balanced response as evidenced by experimental results of 86.33 mg GAE/g DW, 5.93 mg RE/g DW, 87.36% and 55.09% for TPC, TFC, DPPH and ABTS, respectively.

Overall, the natural source of Glu-Gly NaDES can be considered another benefit for the extraction of active substances. Glucose and glycerol are well-known compatible food ingredients that are not toxic. Thus, their mixture is more appropriate as a solvent medium for food-related applications, including nutraceuticals, cosmetics, and pharmaceuticals, since residual toxicity of the solvent is one of the factors. Furthermore, low volatility and biodegradability of these natural components make it possible to conduct a more environmentally friendly extraction process.

### Impact of microwave pretreatment

The microwave pretreatment acts as a process to open up the matrix in order to make the extraction procedure easier through ultrasound (Singla et al. 2023). Microwave heating of the moisture content inside the plant cell quickly leads to the development of internal pressure and heat stress, leading to the weakening of cell wall and membrane structure and thus rendering the tissue more permeable (Maqsood and Ahmed 2025). It helps facilitate the movement of DES inside the matrix and reduces the diffusion path of the intracellular phytochemicals. Once ultrasound is applied, cavitation effects on the already permeated matrix lead to the production of local shear and microturbulence (Vilkhu et al. 2008; Imtiaz et al. 2026). This makes the process complementary because microwave pretreatment enhances the matrix permeation while cavitation of ultrasound increases the speed of releasing solutes into the extracting solvent (Qadeer et al. 2025; Waseem et al. 2026).

### Antimicrobial studies

**Fig. 4:**
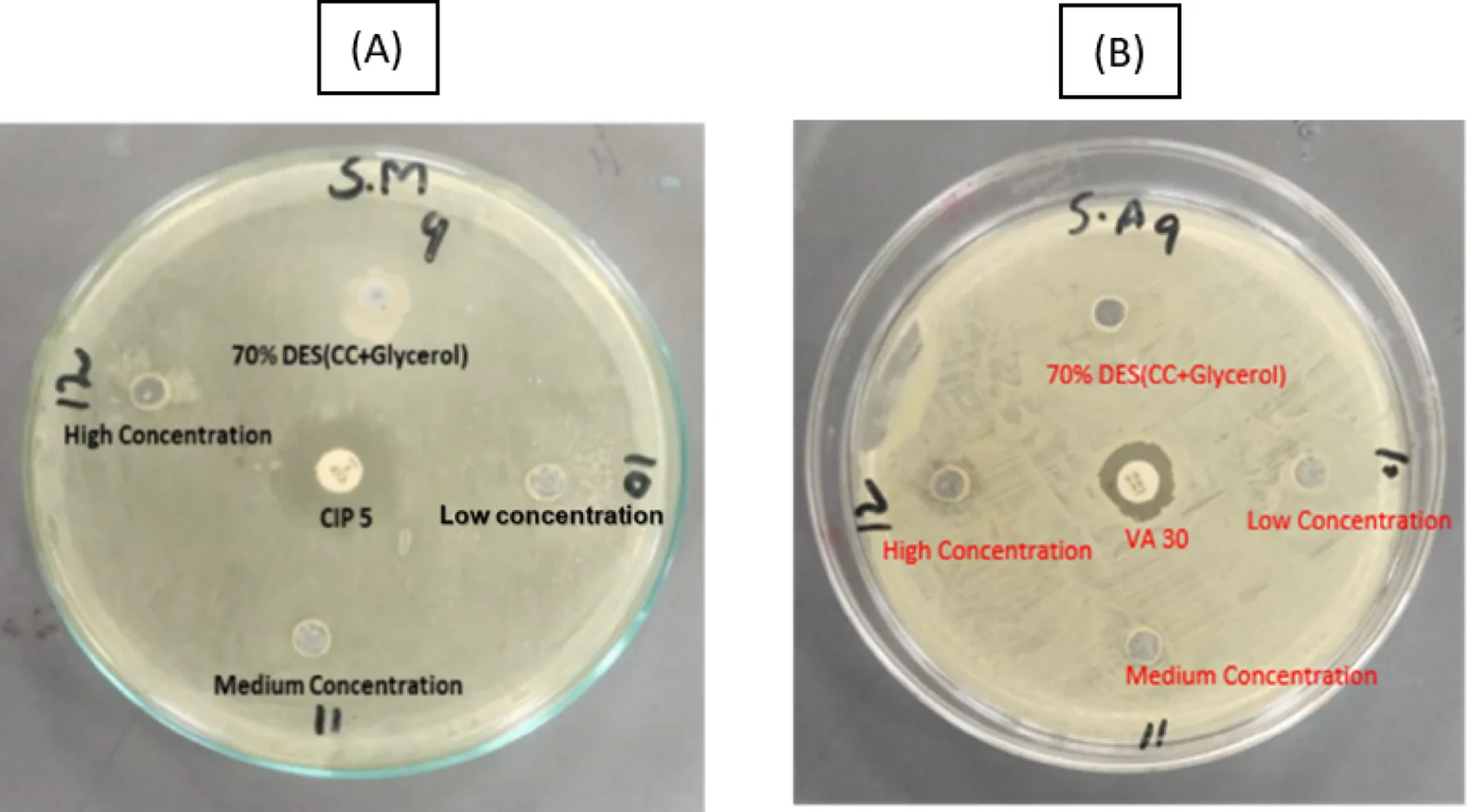
Antibacterial activity of 70% deep eutectic solvent (DES) extracts against *Salmonella typhi* (SM) and *Staphylococcus aureus* (SA) using the agar well diffusion method. The plates illustrate zones of inhibition at high, medium, and low extract concentrations for extracts. Ciprofloxacin (CIP5; **A**) and vancomycin (VA30; **B**) served as the positive control. Differences in inhibition zones reflect the varying antimicrobial potential of the tested DES extracts

The antibacterial activities were evaluated by using agar well diffusion method and zone of inhibition were measured (Table 4; Fig. 4). The antibacterial activity of DES-based extracts was evaluated against *Staphylococcus aureus* and *Salmonella typhi*. The antibacterial efficacy of the DES-based extracts was strongly influenced by the nature of the hydrogen-bond donor used in the DES formulation (Bedair et al. 2024; Pozharitskaya et al. 2024). This sugar-based DES likely provides an optimal hydrogen-bonding network and viscosity that enhances the extraction and stabilization of phenolics, flavonoids, or other antimicrobial phytochemicals. The strong antibacterial activity observed against both Gram-positive and Gram-negative bacteria, suggests efficient extraction of broad-spectrum antibacterial compounds. The gradual reduction in activity with dilution further supports the role of extract concentration in governing antibacterial efficacy (Ramon et al. 2023; Swebocki 2024; Awari et al. 2025).

**Table 4:** Antibacterial activity of different DES formulations at varying concentrations against *Staphylococcus aureus* and *Salmonella typhi* measured by zone of inhibition

DES	Serial dilutions used	*Zone of inhibition* (mm) *Staphylococcus aureus* (SA)	*Zone of imhibition* (mm) *Salmonella typhi* (SM)	Remarks
(Glu+Gly)	Undiluted 70% DES extract (25 mL/g SSR)	21	20	Antibacterial
	1:1	12	11	Antibacterial
	1:2	5	-	No response
	DES (70%) Solvent only	17	16	Antibacterial
	Standard drug	13 (VIN)	19 (CIP)	Antibacterial

The glucose–glycerol DES demonstrated pronounced inhibitory effects against both *Staphylococcus aureus* and *Salmonella typhi*. The undiluted extract prepared using 70% aqueous DES at an SSR of 25 mL/g produced inhibition zones of 21 and 20 mm against *S. aureus* and *S. typhi*, respectively, compared with 13 and 19 mm for the corresponding standard antibiotics. This pronounced activity may be associated with the favorable polarity and hydrogen-bonding characteristics of the glucose–glycerol system, which can facilitate the extraction and stabilization of polar bioactive constituents (Makris and Lalas 2020; Lazović et al. 2024).

Importantly, the experimentally validated numerical optimum of the MP-UAE_DES_ process yielded an extract containing 86.33 mg GAE/g DW TPC and 5.93 mg RE/g DW TFC, demonstrating the substantial recovery of phenolic and flavonoid constituents under the optimized extraction conditions. These phytochemical constituents may contribute to the observed antibacterial activity, as phenolic and flavonoid compounds can interact with bacterial cell membranes and other cellular targets. However, the TPC and TFC values were determined for the optimized extract and were not measured separately for each dilution used in the antimicrobial assay; therefore, a direct quantitative correlation between individual TPC/TFC levels and inhibition-zone diameter cannot be established. Nevertheless, the high TPC and measurable TFC of the optimized extract, together with the concentration-dependent reduction in inhibition upon dilution, support the potential contribution of extracted phenolic and flavonoid constituents to the antibacterial activity. Overall, these findings demonstrate that the glucose–glycerol DES not only served as an effective green extraction medium for recovering bioactive compounds from *C. paradisi* peels but also contributed to the observed antibacterial activity. The antibacterial effect of the DES alone, as evidenced by an inhibition zone of 17 mm, was further enhanced when combined with the *C. paradisi* peel extract, producing an inhibition zone of 21 mm. This enhanced activity suggests that the antibacterial properties of the DES may complement the activity of phytochemicals extracted from the peel, resulting in greater overall antibacterial efficacy. Thus, the glucose–glycerol DES-based extraction system represents a promising green approach for obtaining antibacterial extracts from *C. paradisi* peels, with potential applications in the development of sustainable antimicrobial formulations. Further studies involving replicate assays and MIC and MBC determinations are recommended to quantitatively validate and further characterize this antibacterial potential.

## Conclusion

The present study showed that the glucose-glycerol (Glu–Gly) natural deep eutectic solvent was the most suitable solvent among the tested DES systems for extracting bioactive compounds from *Citrus paradisi* peel. RSM-based numerical optimization identified 74% water-content Aq-DES, 20 min sonication time, and a solvent-to-solid ratio of 35 mL/g as the optimum extraction conditions. Experimental validation under these conditions confirmed the suitability of the developed model for optimizing the extraction process. Under these conditions, the extract contained 86.33 mg GAE/g DW of total phenolics and 5.93 mg RE/g DW of total flavonoids, together with strong DPPH and ABTS radical-scavenging activities of 87.36% and 55.09%, respectively. The optimized extract also showed appreciable antibacterial activity against both *Staphylococcus aureus* and *Salmonella typhi*, indicating that the extracted phytochemicals retained their biological activity. The combination of microwave pretreatment, ultrasound-assisted extraction, and a naturally derived DES therefore offers an efficient way to recover valuable compounds from grapefruit peel. Beyond the extraction performance, this approach gives added value to an abundant citrus-processing residue while reducing dependence on conventional organic solvents. The use of glycerol and glucose as solvent components, together with the recovery of antioxidant and antibacterial compounds, makes the process relevant to the principles of green chemistry and circular economy. Thus, *C. paradisi* peel can be considered a promising low-cost source of functional bioactive extracts rather than simply an agro-industrial waste. Some limitations of the present study should be acknowledged. Individual phenolic constituents were not characterized using HPLC/LC–MS, and the investigation was based on a single batch of plant material. Further studies involving multiple batches and chromatographic profiling are therefore needed to establish the detailed chemical composition and reproducibility of the optimized extract.

## Data Availability

The data supporting the findings of this study are available from the corresponding author upon reasonable request.
